# Simplified Segmentectomy of Right Subsuperior and Posterior Basal Segments

**DOI:** 10.1016/j.atssr.2022.08.003

**Published:** 2022-08-17

**Authors:** Akio Hara, Hideoki Yokouchi

**Affiliations:** 1 Department of Surgery, Suita Municipal Hospital, Suita, Japan

## Abstract

Thoracoscopic segmentectomy, involving the posterior basal segment of the lower lobe, is one of the most challenging procedures and often requires incision of irrelevant lung parenchyma from the interlobar fissure and complex stapling techniques for dividing intersegmental planes. This report describes a simplified thoracoscopic segmentectomy for the subsuperior and posterior basal segments (S∗ + S10), without interlobar fissure dissection or complex intersegmental division.

Thoracoscopic segmentectomy, involving the posterior basal segment of the lower lobe, is a very challenging procedure and often requires incision of irrelevant lung parenchyma from the interlobar fissure (Figure 1A). Thoracoscopic anatomic S10 segmentectomy using a posterior approach without interlobar fissure dissection has been reported,^1^^,^^2^ and it requires complex stapling techniques for dividing intersegmental planes (Figure 1B). Here we report a simplified thoracoscopic segmentectomy for the subsuperior and posterior basal segments (S∗ + S10) without interlobar fissure dissection or complex intersegmental division (Figure 1C).

**Figure 1:**
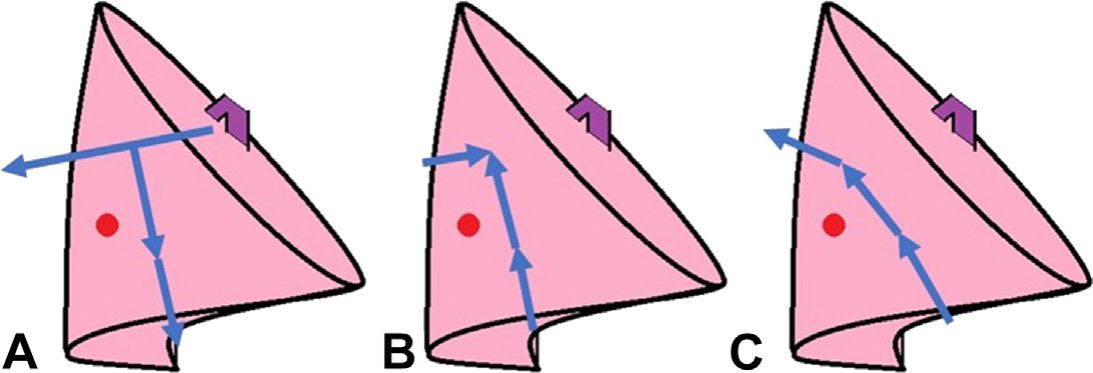
Intersegmental division in posterior basal segmentectomy. (A) Conventional method. (B) Posterior approach. (C) Our simplified method.

## Technique

A 65-year-old woman with multiple ground glass opacity (GGO) nodules in both lungs was referred to our department (Department of Surgery, Suita Municipal Hospital, Suita, Japan). The nodules were observed in S2 and S∗ of the right lung and in S3, S6, S8, S9, and S10 of the left lung. Most nodules were <1 cm in diameter and were considered to be atypical adenomatous hyperplasia or adenocarcinoma in situ (AIS), except for the nodule in the right S∗. The maximum diameter of the S∗ nodule and that of the consolidation were 16 mm and 5 mm, respectively. Positron emission tomography combined with computed tomography showed no lymph node involvement. Thoracoscopic right S∗ + S10 segmentectomy with right S2 wedge resection was performed.

The patient was placed in the left lateral decubitus position with the surgeon on the dorsal side, the assistant operating the thoracoscope on the ventral side, and the monitor on the cranial side. A 7-mm port for the thoracoscope was placed at the midaxillary line of the eighth intercostal space, and a 7-mm port for the left hand of the surgeon was placed at the posterior axillary line of the seventh intercostal space. An access port with a 4-cm skin incision was made anteriorly along the midaxillary line at the sixth intercostal space, and an Alexis Wound Protector/Retractor Small (Applied Medical) was used by the right hand of the surgeon and the assistant.

**Figure 2:**
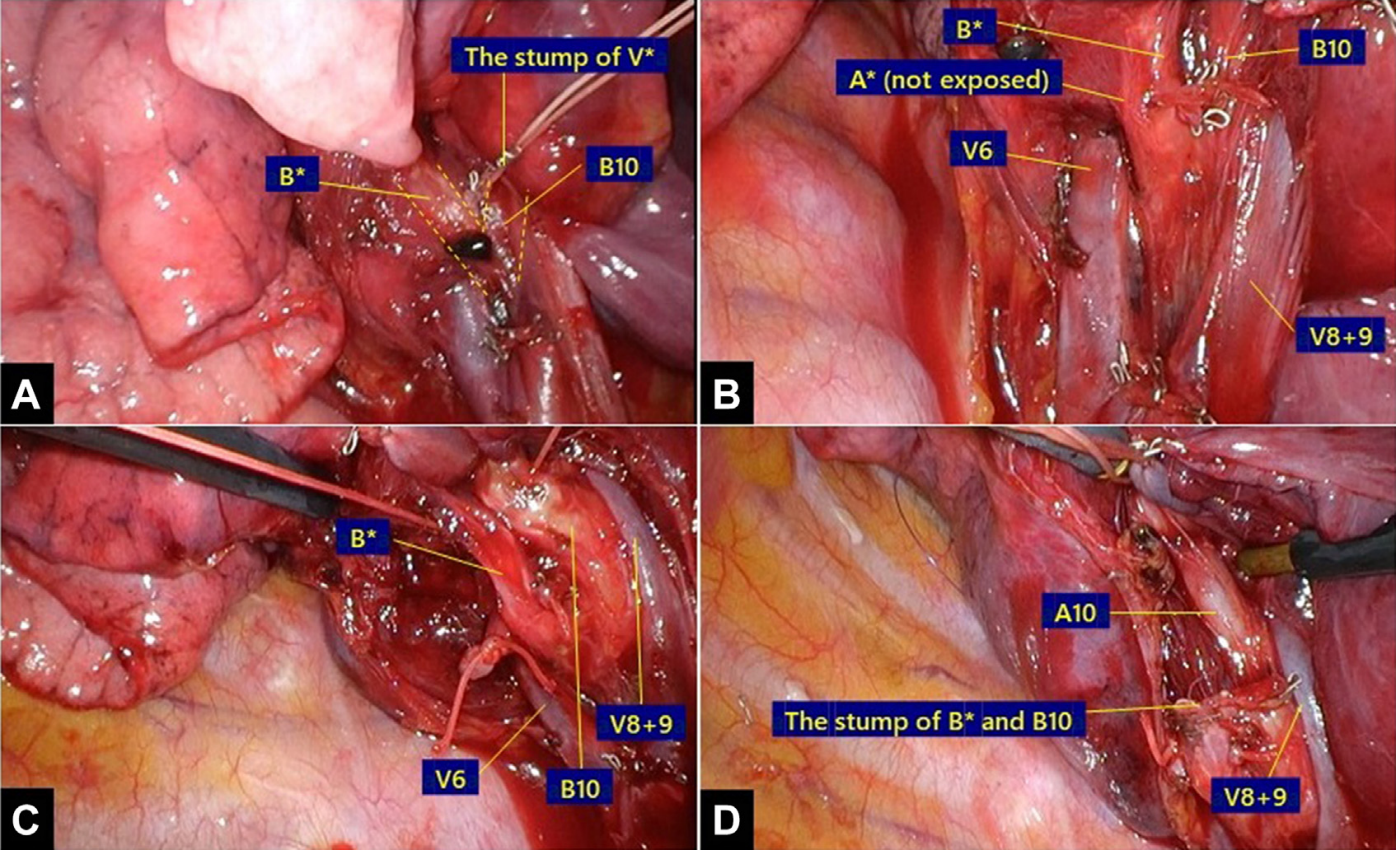
Identification of vessels and bronchi. (A) V∗ flew out from between B∗ and B10. (B) V6 flew into the left atrium, crossing from the cranial side to the dorsal side of A∗ and B∗. (C) B∗ and B10 were taped together. (D) V8 + 9 crossed through the ventral side of A10.

Initially, the pulmonary ligament was incised to expose the inferior pulmonary vein. Each branch of the inferior pulmonary vein was dissected toward the proximal side. Branches from within the area to be resected (V∗ and V10) were cut off using staplers after ligating the proximal side for traction. Lateral traction of the resected vein clarified the bifurcation of B∗ and B10 (Figure 2A). V6, including the intersegmental vein between S6 and S∗, was dissected proximally and crossed with A∗ and B∗ (Figure 2B). Similarly, V8 + 9, including the intersegmental vein between S9 and S10, crossed B10 and A10 (Figures 2B-2D). This helped to clarify the anatomic structure by using a posterior approach. A∗ and A10 were ligated and cut. B∗ and B10 were cut together using a stapler (Figure 2C).

**Figure 3:**
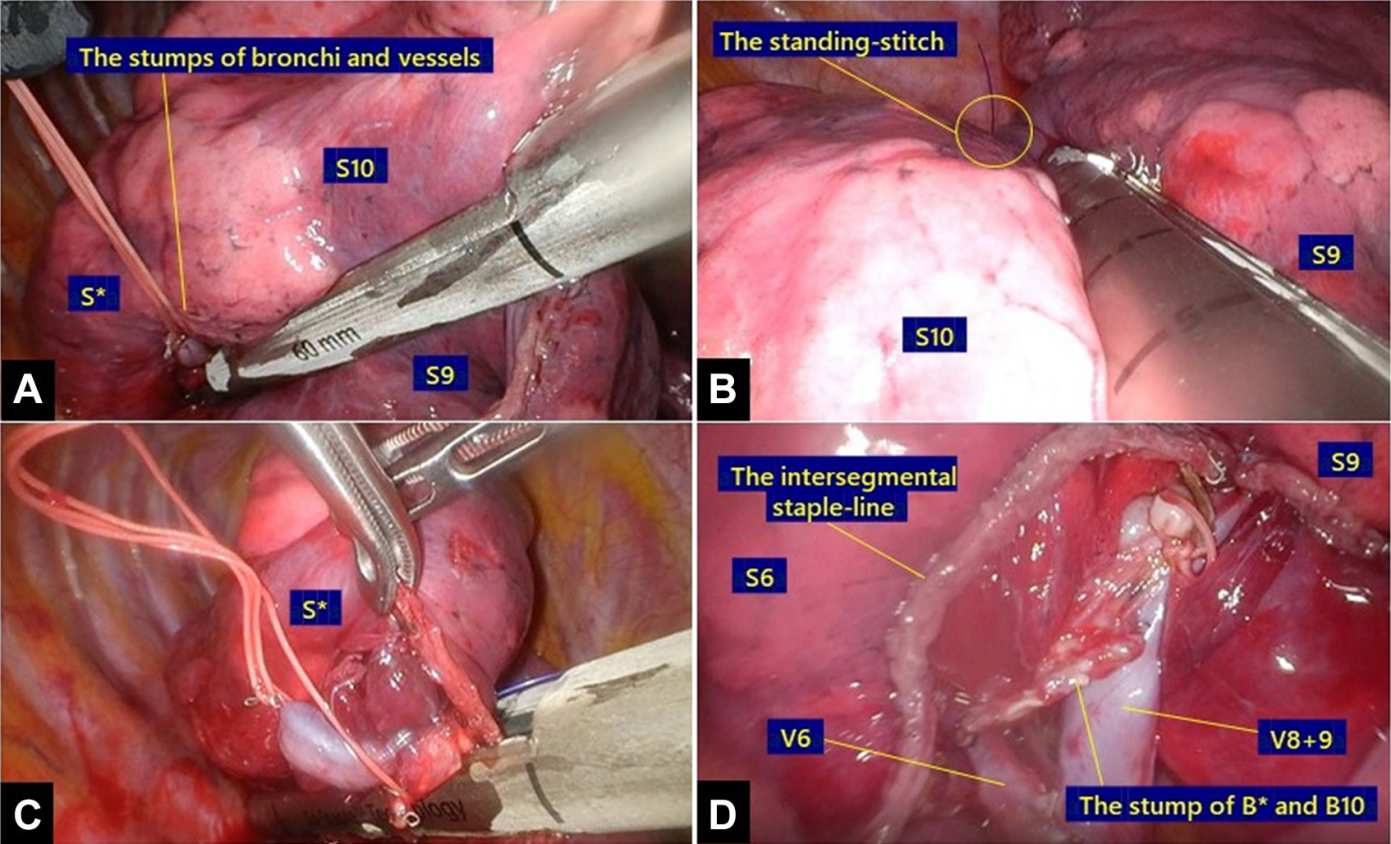
The lung parenchyma was divided using staplers (A) toward the stumps of the segmental bronchi and vessels and (B) toward the standing stitch. (C) Afterward, staplers were inserted in the same direction, to run over the residual lung parenchyma. (D) No intersections were seen on the intersegmental staple line.

Tumor location was identified by finger palpation through the access port, and the “standing stiches” with 4-0 polypropylene sutures (Prolene, Ethicon) were placed along the resection lines, similar to the technique reported by Sato and colleagues,^3^ but with the location modified to the ventral margin of the tumor, instead of at the corner of the intersegmental planes. Staplers were used to divide the lung parenchyma directly toward the standing stitch and stumps of the segmental bronchi and vessels, rather than along anatomically precise intersegmental planes (Figure 3).

Subsequently, right S2 wedge resection was performed after S2 tumor identification. Surgical margins were checked by histopathologic examination of the intraoperative frozen section specimen. Hilar and mediastinal lymph node dissection was not performed.

To prevent air leakage from the intersegmental plane, a low-voltage coagulation system with fibrin glue and a polyglycolic acid sheet were used around the edge of the staple line. The chest tube was placed through the thoracoscope port. The incisions were closed after checking for active bleeding and massive air leakage.

The operative time was 242 minutes, and the amount of surgical blood loss was 70 mL. Air leakage from the chest drain was stopped after extubation. Subsequently, the drain was removed on postoperative day 3. The patient was discharged on postoperative day 11 without any complications. Pathologic findings established the diagnosis in both nodules with AIS and revealed no tumor involvement at the cut end. Postoperative pulmonary function tests at 6 months postoperatively showed only a 6% decrease from the preoperative findings (vital capacity, 2380-2310 mL; forced expiratory volume in 1 second, 17001600 mL). Pulmonary function fully recovered at 12 months postoperatively (vital capacity, 2430 mL; forced expiratory volume in 1 second, 1720 mL).

## Comment

Multiple GGOs suggestive of early-stage lung cancer are often treated by wedge resections or segmentectomies, if they cannot be resected in their entirety by lobectomy. However, local recurrence or tumor progression in lesions other than those resected may require complete lobectomy or ipsilateral lobectomy of the other lobe. Because of the future risk of requiring ipsilateral reoperations, interlobar pulmonary artery exposure should ideally be avoided to prevent adhesion of the interlobar fissure.

Segmentectomy may be considered for GGOs suggestive of AIS or minimally invasive adenocarcinoma if wedge resection is difficult because of tumor size or location. In general, anatomically precise segmentectomy is performed to account for adequate lymphatic drainage. However, previous observational studies reported no significant differences in recurrence-free survival between wedge resection and segmentectomy for GGO-dominant early-stage lung cancer.^4^^,^^5^ Therefore, we thought that the key to reducing local recurrence would not be anatomic accuracy in the intersegmental planes, but rather adequate resection of the lung parenchyma margin. There are no major concerns regarding resection lines of the lung parenchyma dislocating into adjacent segments.

Complex segmentectomy for a segment having >1 intersegmental plane may be associated with postoperative pulmonary complications,^6^ such as air leakage, which may be reduced by dividing the lung parenchyma as simply as possible using staplers. The disadvantages of this procedure are the possibility of atelectasis in adjacent areas by bronchiole stenosis and the shortened resection margin in the deep lung parenchyma by stapling. However, these complications may be avoided by setting the resection lines to cross nearly vertically with the bronchioles and by using techniques such as the standing stitch, which allows visualization of the resection margins.^3^

The concept of our technique is a wide wedge resection with separation of segmental bronchi and vessels. This technique, which resembles simple segmentectomy rather than complex segmentectomy, may reduce postoperative pulmonary complications and may be useful in cases with a potential risk of ipsilateral reoperation.
